# Kondo scattering in underdoped Nd_1−x_Sr_x_NiO_2_ infinite-layer superconducting thin films

**DOI:** 10.1093/nsr/nwad112

**Published:** 2023-04-25

**Authors:** Ting-Na Shao, Zi-Tao Zhang, Yu-Jie Qiao, Qiang Zhao, Hai-Wen Liu, Xin-Xiang Chen, Wei-Min Jiang, Chun-Li Yao, Xing-Yu Chen, Mei-Hui Chen, Rui-Fen Dou, Chang-Min Xiong, Guang-Ming Zhang, Yi-Feng Yang, Jia-Cai Nie

**Affiliations:** Department of Physics, Beijing Normal University, Beijing100875, China; Department of Physics, Beijing Normal University, Beijing100875, China; Department of Physics, Beijing Normal University, Beijing100875, China; Department of Physics, Beijing Normal University, Beijing100875, China; Department of Physics, Beijing Normal University, Beijing100875, China; Department of Physics, Beijing Normal University, Beijing100875, China; Department of Physics, Beijing Normal University, Beijing100875, China; Department of Physics, Beijing Normal University, Beijing100875, China; Department of Physics, Beijing Normal University, Beijing100875, China; Department of Physics, Beijing Normal University, Beijing100875, China; Department of Physics, Beijing Normal University, Beijing100875, China; Department of Physics, Beijing Normal University, Beijing100875, China; State Key Laboratory of Low-Dimensional Quantum Physics and Department of Physics, Tsinghua University, Beijing100084, China; Frontier Science Center for Quantum Information, Beijing100084, China; Beijing National Laboratory for Condensed Matter Physics and Institute of Physics, Chinese Academy of Sciences, Beijing100190, China; School of Physical Sciences, University of Chinese Academy of Sciences, Beijing100190, China; Songshan Lake Materials Laboratory, Dongguan523808, China; Department of Physics, Beijing Normal University, Beijing100875, China

**Keywords:** Kondo scattering, superconductivity, underdoped, infinite-layer nickelate, thin film

## Abstract

The recent discovery of superconductivity in infinite-layer nickelates generates tremendous research endeavors, but the ground state of their parent compounds is still under debate. Here, we report experimental evidence for the dominant role of Kondo scattering in the underdoped Nd_1−x_Sr_x_NiO_2_ thin films. A resistivity minimum associated with logarithmic temperature dependence in both longitudinal and Hall resistivities are observed in the underdoped Nd_1−x_Sr_x_NiO_2_ samples before the superconducting transition. At lower temperatures down to 0.04 K, the resistivities become saturated, following the prediction of the Kondo model. A linear scaling behavior $\sigma _{{\boldsymbol{xy}}}^{{{\bf AHE}}}{\mathrm{\ }}\sim{\mathrm{\ }}{\sigma }_{{\boldsymbol{xx}}}$ between anomalous Hall conductivity $\sigma _{{\boldsymbol{xy}}}^{{\bf{AHE}}}$ and conductivity ${\sigma }_{{\boldsymbol{xx}}}{\mathrm{\ }}$is revealed, verifying the dominant Kondo scattering at low temperature. The effect of weak (anti-)localization is found to be secondary. Our experiments can help in clarifying the basic physics in the underdoped Nd_1−x_Sr_x_NiO_2_ infinite-layer thin films.

## Teaser

Experimental evidences for the dominant role of Kondo scattering were discovered in the underdoped Nd_1−x_Sr_x_NiO_2_ infinite layer superconducting thin films.

## INTRODUCTION

The mechanism of high-*T*_c_ superconductivity remains a long-standing mystery, even though tremendous efforts and significant advances have been made in the study of cuprates and Fe-based superconductors. Aiming to mimic the cuprate-like electronic configuration, superconductivity has been proposed and recently found in the infinite-layer nickelate compounds [1]. This discovery has attracted great attention [2–6] as it may provide deeper insights into the pairing mechanism of unconventional superconductivity. But instead of an antiferromagnetic (AFM) order in the cuprates, the parent compound of superconducting nickelates shows no sign of static long-range magnetic order down to 1.7 K by neutron diffraction [7] and down to 2 K by muon spin measurements [8] in bulks and also a lack of long-range AFM in thin films [9], despite their similarities in the crystalline and electronic structures with the formal $3{{\mathrm{d}}}^9$configuration. Moreover, unlike the cuprates where doped holes reside on the oxygen atoms, here they are mainly introduced into Ni $3{\mathrm{d}}$ states and reside in the $3{{\mathrm{d}}}_{{{\mathrm{x}}}^2 - {{\mathrm{y}}}^2}$ orbitals [10], supported by the softening of resonant inelastic X-ray scattering (RIXS) [11]. Besides, superconductivity remains absent in infinite-layer nickelate bulk, although the application of pressure up to 50.2 GPa can significantly suppress the insulating behavior [12,13]. This raises a question regarding whether the cuprates and hole doped NdNiO_2_ share the same superconductivity mechanism [14]. In the experimental aspect, controlling doping concentration and studying its relationship with *T*_c_ has become a necessary step to understand the electron interaction in nickelate systems. A non-monotonic relationship between *T*_c_ and Sr doping concentration is established [3,15], reminiscent of the competition between superconductivity and adjacent phase (or its phase fluctuation) in unconventional superconductors [16–18]. It is therefore important to understand the underdoped nickelates, where, different from the cuprates, the resistivity shows metallicity at high temperatures and weak insulating behavior at low temperatures.

In order to explain this upturn in resistivity at low temperatures, Yang and Zhang [19] proposed a self-doped Mott–Kondo scenario for the parent nickelate system, bridging the Kondo lattice model for heavy fermions and the *t–J* model for cuprates. It was proposed that self-doping, namely charge transfer from localized Ni 3d orbitals to other conduction bands, plays an essential role. The remaining Ni 3d local moments may couple to the conduction electrons, causing the well-known Kondo screening physics [20] and giving rise to Kondo scattering that explains the low temperature resistivity upturn reported in NdNiO_2_ [1], LaNiO_2_ [9], as well as underdoped infinite-layer Nd_1−x_Sr_x_NiO_2_ (x = 0.1, 0.125) [15]. The absence of long-range magnetic ordering [9] might also be attributed to the self-doping and the Kondo screening effect. However, in addition to other theoretical scenarios including the pseudogap [21], magnetic scattering [22] and the *d*-wave order [23], the logarithmic temperature dependence of resistivity at low temperatures has also been observed in underdoped cuprates and was mainly attributed to weak localization (WL) [24–26]. Although it has been argued theoretically [5,27] that the transport properties of the underdoped nickelates are very different from that of the AFM cuprate Mott insulator, whether the lnT behavior in the underdoped nickelates is caused by the Kondo effect or by the WL effect has not been determined, which leads to unresolved debate concerning the basic physics of nickelate superconductors. The transport measurements, including both longitudinal and transverse resistivity, are therefore indispensable to uncover the low-energy excitations of the parent compounds of nickelate superconductor and provide conclusive evidence for different theoretical proposals in order to understand the unconventional superconductivity in the nickelate system.

Here, we study the normal-state transport properties of the infinite-layer Nd_1−x_Sr_x_NiO_2_ thin films with a low Sr doping concentration. Our preliminary analysis of the film reveals a lnT behavior in both resistivity and *R_H_* in the same temperature region. At lower temperatures down to 0.04 K, the resistivity becomes saturated. Good agreement between the experimental data and the theoretical prediction of the Kondo scenario provides conclusive evidence for the Kondo mechanism. Moreover, a linear dependence of the anomalous Hall-effect (AHE) conductance $\sigma _{{\mathrm{xy}}}^{{\mathrm{AHE}}}{\mathrm{\ }}\sim{\mathrm{\ }}{\sigma }_{{\mathrm{xx}}}$ indicates that Kondo scattering plays a dominant role in the corresponding temperature range. The electron dephasing rate deduced from the magnetoresistance data shows a linear temperature dependence below the Kondo temperature (T_K_), in good agreement with the Kondo scenario. Last, careful analysis demonstrates that the effect of weak (anti-)localization plays only a secondary role. Our experimental results strongly support the self-doped Mott–Kondo scenario for the underdoped Nd_1−x_Sr_x_NiO_2_ infinite-layer superconducting thin films.

## RESULTS

**Figure 1.**
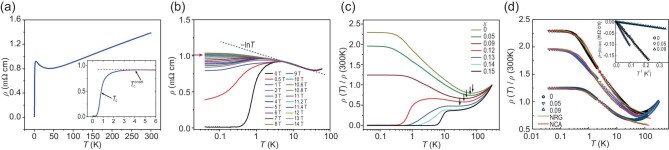
Temperature-dependent resistivity *ρ(T)* for the underdoped Nd_1−x_Sr_x_NiO_2_ thin films. (a) The temperature-dependent resistivity of the Nd_0.88_Sr_0.12_NiO_2_ film at zero magnetic field. ${T}_c$ and $T_c^{onset}$, marked by black arrows, are 0.79 K and 4.06 K, respectively. The inset shows the determination of $T_c^{onset}$. (b) Isomagnetic *ρ(T)* curves of the Nd_0.88_Sr_0.12_NiO_2_ film measured in different applied magnetic field *H*. (c) The zero-field temperature dependence of resistivity of the underdoped Nd_1−x_Sr_x_NiO_2_ films with a Sr doping level *x* from 0.00 to 0.15. The arrows indicate the corresponding resistivity minima. (d) The NRG (green) and NCA (red) fits are shown for the underdoped samples with *x* = 0, 0.05 and 0.09, and *T_K_* = 3.5 K, 3.5 K and 5.5 K, respectively (see details in Fig. S4). Inset in (d) shows T^2^ behavior at the lowest temperatures for the underdoped samples. See online supplementary material for a colour version of this figure.

Figure 1a shows the temperature-dependent resistivity ρ(T) for the underdoped Nd_0.88_Sr_0.12_NiO_2_ thin film. A superconducting transition is observed, with an onset at 4.06 K and a midpoint at 0.79 K. The broad transition indicates the inhomogeneity of the infinite-layer thin film. These observations reveal that the infinite-layer nickelate phase (the structural characterizations are shown in Supplementary Figs S1–S2), not the reduced secondary phase, is superconducting. As shown in Fig. 1a, the resistivity of the Nd_0.88_Sr_0.12_NiO_2_ thin film first decreases as the temperature decreases, followed by a resistivity minimum at a characteristic temperature T*∼ 40 K. The minimum resistivity (∼0.80 mΩ⋅cm at 40 K) falls below the value of 0.87 mΩ⋅cm that corresponds to the quantum sheet resistance (h/e^2^∼26 kΩ) per NiO_2_ two-dimensional plane, which is consistent with the previous report [15]. Below 30 K down to 7 K, the resistivity shows logarithmic temperature dependence regardless of the magnetic field (see Fig. 1b). In fact, this characteristic is commonly observed in all our underdoped Nd_1−x_Sr_x_NiO_2_ infinite-layer thin films, as shown in Fig. 1c. We find that the ρ–T curves can be well described by the Hamann model from 8 K to 120 K [28],


(1) \begin{eqnarray*}\!\!\!\!{\mathrm{\rho }}\left( {\mathrm{T}} \right) = {{\mathrm{\rho }}}_0{\mathrm{\ }} + {\mathrm{a}}{{\mathrm{T}}}^2 + {\mathrm{b}}{{\mathrm{T}}}^5 + {{\mathrm{\rho }}}_{\mathrm{K}}\left( {{\mathrm{T}}/{{\mathrm{T}}}_{\mathrm{K}}} \right),\end{eqnarray*}

where T_K_ is the Kondo temperature, ${{\mathrm{\rho }}}_0$ is the residual resistivity caused by sample disorder and the ${{\mathrm{T}}}^2$ and ${{\mathrm{T}}}^5$ terms are the contributions of electron-electron and phonon-electron interactions, respectively. ${{\mathrm{\rho }}}_{\mathrm{K}}( {{\mathrm{T}}/{{\mathrm{T}}}_{\mathrm{K}}} )$ is the resistivity induced by magnetic scattering in the absence of magnetic field [28,29] and takes the form,


(2) \begin{eqnarray*}{{\mathrm{\rho }}}_{\mathrm{K}}\left( {{\mathrm{T}}/{{\mathrm{T}}}_{\mathrm{K}}} \right) &=& \frac{{2{\mathrm{\pi c}}\hbar }}{{{\mathrm{n}}{{\mathrm{e}}}^2{{\mathrm{k}}}_{\mathrm{F}}}}\\
&& \left\{ 1 - {\mathrm{ln}}\left( {{\mathrm{T}}/{{\mathrm{T}}}_{\mathrm{K}}} \right) \cdot \left[ {\mathrm{l}}{{\mathrm{n}}}^2\left( {{\mathrm{T}}/{{\mathrm{T}}}_{\mathrm{K}}} \right)\right.\right.\\
&&\left.\left. +\ {\mathrm{s}}\left( {{\mathrm{s}} + 1} \right){{\mathrm{\pi }}}^2 \right]^{ - 1/2} \right\},
\end{eqnarray*}

where ${{\mathrm{k}}}_{\mathrm{F}} = {\mathrm{\ }}2{\mathrm{\pi }}{( {\frac{{3{\mathrm{n}}}}{{8{\mathrm{\pi }}}}} )}^{1/3}$ is the Fermi wave-vector in a free electron approximation. The best fitting results to the ρ–T curves of underdoped Nd_1−x_Sr_x_NiO_2_ samples using Eqs. (1) and (2) are shown in Supplementary Fig. S5. Moreover, we have carried out low-temperature transport measurements in the magnetic field for three underdoped Nd_1−x_Sr_x_NiO_2_ samples (*x* = 0, 0.05, 0.09). All three underdoped samples are well consistent with the Kondo scattering scenario [20] down to 0.04 K. The numerical renormalization group (NRG) fitting, non-crossing approximation (NCA) fitting (see Fig. 1d and Supplementary Fig. S4) and Hamann fitting (Supplementary Fig. S5) curves provide quantitative justification for this point. The magnetic field truly influences the *R–T* curves of underdoped samples with pronounced negative magnetoresistance (as shown in Supplementary Fig. S5), reminiscent of the well-known negative magnetoresistance in the Kondo system (La, Ce)Al_2_ [30]. Moreover, all the underdoped samples (Fig. 1d) demonstrate clear features of Kondo scattering in temperature regime above ${T}_K$. For temperatures below ∼0.6 K, the resistivity of the underdoped samples clearly follows a T^2^ temperature dependence (inset of Fig. 1d), as expected for Kondo-like behavior [31,32]. The good agreement with the NRG, NCA and Hamann predictions proves exclusively the Kondo mechanism and leaves little room for alternative interpretation in the underdoped region, which also confirms that the local moment is roughly spin-1/2. This excludes its possible origin from the Nd 4f spin (S = 3/2) and supports its origin from the Ni $3{{\mathrm{d}}}_{{{\mathrm{x}}}^2 - {{\mathrm{y}}}^2}$ moment.

## DISCUSSION

**Figure 2.**
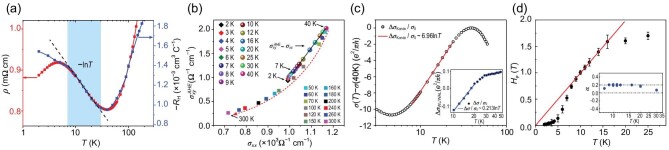
Scaling behavior of anomalous Hall conductivity and conductance corrections for Nd_0.88_Sr_0.12_NiO_2_. (a) Logarithmic temperature dependence of the resistivity (red) and the Hall coefficient (blue) of the Nd_0.88_Sr_0.12_NiO_2_ film. The light cyan area represents the Kondo region. (b) Plot of AHE conductivity $\sigma _{xy}^{\rm AHE}$ vs conductivity ${\sigma }_{xx}$ of the Nd_0.88_Sr_0.12_NiO_2_ film over the entire temperature range. Since ${\rho }_{xy} \sim {\rho }_{xx}/{10}^3$, we can simplify the anomalous Hall conductivity as $\sigma _{xy}^{\rm AHE} = \ - \rho _{xy}^{\rm AHE}/\rho _{xx}^2,{\mathrm{\ here\ }}\rho _{xy}^{\rm AHE} \equiv ( {{R}_H - {R}_0} ) \cdot H,{\mathrm{\ }}$and the longitudinal conductivity as ${\sigma }_{xx} = 1/{\rho }_{xx}\ $. A linear dependence (solid black line) of $\sigma _{xy}^{\rm AHE}\ \sim\ {\sigma }_{xx}$ is obvious in a temperature range of 7−40 K. (c) Zero field conductance correction for the Nd_0.88_Sr_0.12_NiO_2_ film due to the Kondo effect, i.e. $\Delta \ {\sigma }_{Kondo} = \ \sigma ( T ) - \sigma ( {\ {T}_{\min} = \ 40K} )$. Data are extracted from Fig. 1b and the solid red line is the ${\mathrm{lnT}}$ fits. Inset: Conduction correction due to the WL/WAL effect, which is obtained with $\Delta \ {\sigma }_{WL/WAL} = \sigma ( T ){|}_{H = 0}\ \ - \ \sigma ( T ){|}_{H = 4T}$ (solid circles). The solid blue line is the $lnT$ fit for obtaining *Δκ = αp*, and here ${\sigma }_0 = {e}^2/\pi h\ $. (d) Dephasing field ${H}_\varphi $ versus temperature for the Nd_0.88_Sr_0.12_NiO_2_ film. The straight red line is the linear fit. The inset shows the corresponding α values, which are nearly constant in the temperature range of 9−20 K. See online supplementary material for a colour version of this figure.

**Figure 3.**
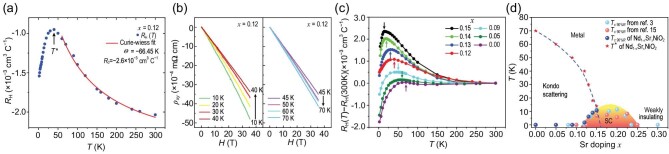
Kondo scattering dominated region in phase diagram of Nd_1−x_Sr_x_NiO_2_. (a) Hall coefficient *R_H_(T*) acquired in a field of 9 T for the Nd_0.88_Sr_0.12_NiO_2_ film with a maximum around 40 K. (b) Measured Hall resistivity, *ρ_xy_*, versus magnetic field, *H*, at different temperatures. The Hall resistivity *ρ_xy_* shows no obvious deviation from linear dependence on magnetic field *H* up to 35 T at all temperatures. (c) Hall coefficient *R_H_*(*T *)*−R_H_*(*300* *K*) acquired in a field of 9 T for the underdoped Nd_1−x_Sr_x_NiO_2_ films, with a maximum around T*, at which the resistivity normally shows a minimum (see the arrows in Fig. 1c). The corresponding characteristic temperatures T* are indicated by the arrows. (d) The superconducting transition temperature *T*_c_ (circles) and the characteristic temperature T* (stars) where the *R_H_* shows a maximum (Fig. 3c). The *T*_c90%R_ is defined to be the temperature at which the resistivity drops to 90% of the value at the onset of the superconductivity. The cyan and orange circles represent the average *T*_c90%R_ adapted from references [3,15]. The blue circles represent the *T*_c90%R_ of the samples shown in this study.

Remarkably, for temperatures below 40 K down to 6 K, the *R_H_* follows closely that of the resistivity ρ, and resembles the lnT dependence of the resistivity, as shown in Fig. 2a (please note the sign, when the temperature decreases in the regime below 40 K, |*R_H_*| increases rather than decreases). The ${{\mathrm{R}}}_{\mathrm{H}} \propto \rho $ behavior is well consistent with the theoretical prediction of Kondo skew scattering associated with local moments [33,34]. Thus, both the resistivity and Hall coefficient support the presence of magnetic Kondo scattering in the underdoped nickelate superconductor. The relation between $\sigma _{{\mathrm{xy}}}^{{\mathrm{AHE}}}$ and ${\sigma }_{{\mathrm{xx}}}$ can further confirm the skew scattering mechanism characterized by $\sigma _{{\mathrm{xy}}}^{{\mathrm{AHE}}} \propto {\sigma }_{{\mathrm{xx}}}$ in a temperature range of 7−40 K for the Nd_0.88_Sr_0.12_NiO_2_ thin film (see Fig. 2b). The conductivity ${\sigma }_{{\mathrm{xx}}}$ of the Nd_0.88_Sr_0.12_NiO_2_ is about 10^3^ Ω^−1^ cm^−1^ and is in a bad metal regime [34], in which $\sigma _{{\mathrm{xy}}}^{{\mathrm{AHE}}}$ should generally decrease with decreasing ${\sigma }_{{\mathrm{xx}}}$ at a rate faster than linear. Nevertheless, by subtracting the ordinary Hall effect (OHE) contribution (R_0_, which is determined by the measured Hall coefficients and the Curie–Weiss fit and does not change with varying temperature, see detailed discussion in Eqs. (4) and (5) and results in Fig. 3a) to obtain $\sigma _{{\mathrm{xy}}}^{{\mathrm{AHE}}}$ from ${\sigma }_{{\mathrm{xy}}}$, a linear dependence of $\sigma _{{\mathrm{xy}}}^{{\mathrm{AHE}}} \propto {\sigma }_{{\mathrm{xx}}}{\mathrm{\ }}$is observed in the temperature range of 7−40 K. This temperature range is the same as the range where resistivity shows logarithmic temperature dependence (see Fig. 2a). These observations strongly support that the incoherent skew scattering [33,34] is a dominant mechanism for understanding our *R_H_(T)* results. At high temperatures (>40 K), the Kondo scattering is suppressed and a deviation from the linear dependence between $\sigma _{{\mathrm{xy}}}^{{\mathrm{AHE}}}$ and ${\sigma }_{{\mathrm{xx}}}$ is also observed.

One may argue that the logarithmic temperature dependence of resistivity at low temperatures, a hallmark of the Kondo effect, could also originate from the weak localization/weak anti-localization (WL/WAL) in a two-dimensional system. However, as shown in Fig. 1b, the lnT correction of resistivity under a large magnetic field can exclude the WL/WAL correction and justify the Kondo scattering scenario. The conductance correction due to the Kondo effect can be obtained with ${\mathrm{\Delta \ }}{{\mathrm{\sigma }}}_{{\mathrm{Kondo}}} = {\mathrm{\ \sigma }}( {\mathrm{T}} ) - {\mathrm{\sigma }}( {\ {{\mathrm{T}}}_{{\mathrm{min}}} = {\mathrm{\ }}40{\mathrm{K}}} )$ (zero field) and the data (extracted from Fig. 1b) are shown in Fig. 3a. The red line is the ${{\mathrm{e}}}^2/{\mathrm{\pi h}} \cdot {\mathrm{lnT}}$ fit for ${\mathrm{\Delta \sigma }}$, the obtained slope is β ≈ 6.96 for the temperature range of 8–24 K. Although both the WL/WAL and the electron-electron interaction (EEI) in a two-dimensional system also contributes to a conductance correction proportional to ${{\mathrm{e}}}^2/{\mathrm{\pi h}} \cdot {\mathrm{lnT}}$, the total value for the coefficient of WL/WAL and EEI corrections is commonly less than 2 [35]. Thus, the value of β is much larger than the typical value contributed from WL/WAL and EEI effect (see Supplementary Note 1). In order to single out the WL/WAL correction, the measurement in a modest magnetic field (e.g. H = 4 T) is necessary (Supplementary Fig. S8). The conduction correction due to the WL/WAL effect can be obtained with ${\mathrm{\Delta \ }}{{\mathrm{\sigma }}}_{{\mathrm{WL}}/{\mathrm{WAL}}} = {\mathrm{\sigma }}( {\mathrm{T}} ){|}_{{\mathrm{H}} = 0}{\mathrm{\ \ }} - {\mathrm{\ \sigma }}( {\mathrm{T}} ){|}_{{\mathrm{H}} = 4{\mathrm{T}}}$, and the data are shown in the inset of Fig. 2c. The blue line is the ${\mathrm{lnT}}$ fit, the obtained slope is Δκ = *αp* ≈ 0.213 (*p* is the dephasing exponent and denotes the strength of dephasing rate versus temperature, ${\mathrm{\tau }}_{\mathrm{\varphi }}^{ - 1}{\mathrm{\ }}\sim{\mathrm{\ }}{{\mathrm{T}}}^{\mathrm{p}}$ [35]) for the temperature range of 10−20 K, much less than the total value β ≈ 6.96, indicating that the WL/WAL is a secondary effect and Kondo scattering is dominant in the temperature range of 7−30 K. See online supplementary material for a colour version of this figure.

Moreover, we can also utilize the low field magneto-conductivity to analyze the temperature dependence of electron dephasing, based on the modified Hikami–Larkin–Nagaoka (HLN) formula [36]:


(3) \begin{eqnarray*}\Delta {\mathrm{\sigma }}\left( {\mathrm{H}} \right) = \frac{{{\mathrm{\alpha }}{{\mathrm{e}}}^2}}{{{\mathrm{\pi h}}}}\ \left[ {{\mathrm{\Psi }}\left( {\frac{1}{2} + \frac{{{{\mathrm{H}}}_{\mathrm{\varphi }}}}{{\mathrm{H}}}} \right) - {\mathrm{ln}}\frac{{{{\mathrm{H}}}_{\mathrm{\varphi }}}}{{\mathrm{H}}}} \right],\end{eqnarray*}

where ${\mathrm{\Psi }}$ is the digamma function, ${{\mathrm{H}}}_{\mathrm{\varphi }} = \hbar /4{\mathrm{eD}}{\tau }_\varphi$, ${\mathrm{D}}$ is the electronic diffusion constant, and ${\mathrm{\alpha }}$ is an effective constant depending on the relative strengths of magnetic scattering and spin-orbit coupling (see Supplementary Figs S8–S9 for related results and see Supplementary Figs S10–S14 for detailed analysis). As shown in the inset of Fig. 2d, for the nickelate thin film studied this work, a small positive value α ≈ 0.20 can be maintained for a wide temperature range of 9−20 K. As the dephasing rate ${\mathrm{\tau }}_{\mathrm{\varphi }}^{ - 1}$ is simply proportional to ${{\mathrm{H}}}_{\mathrm{\varphi }}$, there exists a linear power-law dependence: ${{\mathrm{H}}}_{\mathrm{\varphi }}\sim{\mathrm{\ }}{{\mathrm{T}}}^{\mathrm{p}}$ with p ≈ 1. Indeed, as shown in Fig. 2d, a linear temperature dependence of the dephasing field ${{\mathrm{H}}}_{\mathrm{\varphi }}$ is observed over a wide temperature range of 8−16 K below T_K_, which is consistent with the universal dephasing rate due to Kondo impurities [37]. At temperatures below 8 K, the deviation of *τ_ϕ_*^−1^ ∝ *T* might originate from the influence of superconductivity fluctuation. The dephasing field ${{\mathrm{H}}}_{\mathrm{\varphi }}$ also exhibits a deviation from the linearity around ∼16.0 K. This is due to resonant Kondo scattering in the vicinity of the Kondo temperature and is another definition of T_K_ [38,39]. The obtained value of T_K_ (∼16.0 K) is well consistent with that (∼15.6 K, see Supplementary Fig. S5d) determined by the resistivity measurements. Thus, the Kondo scenario provides a consistent explanation of all our measured data.

Figure 3a displays the Hall-effect measurements obtained in large temperature range under an applied field of 9 T, showing negative Hall coefficients (*R_H_*) with a maximum at T*∼40 K. Interestingly, we find the Hall coefficients now follow a simple Curie–Weiss law. To see this, we separate the normal coefficient ${{\mathrm{R}}}_0$ from the AHE coefficient and make the ansatz based on the treatment of heavy fermion superconductors [40]


(4) \begin{eqnarray*}{{\mathrm{\rho }}}_{{\mathrm{xy}}} = {{\mathrm{R}}}_0{\mathrm{\ H}} + 4{\mathrm{\pi M}}{{\mathrm{R}}}_{\mathrm{S}}.\end{eqnarray*}

Taking M = χH, χ = C/(T−Θ), where M is the magnetization, χ is the magnetic susceptibility, C is the Curie constant and Θ is the Curie temperature, we have


(5) \begin{eqnarray*}{{\mathrm{R}}}_{\mathrm{H}} = \frac{{{{\mathrm{\rho }}}_{{\mathrm{xy}}}}}{{\mathrm{H}}} = {{\mathrm{R}}}_0{\mathrm{\ }}\! +\! 4{\mathrm{\pi }}\frac{{\mathrm{C}}}{{{\mathrm{T}}\! -\! \Theta }}\ {{\mathrm{R}}}_{\mathrm{S}}\! =\! {{\mathrm{R}}}_0{\mathrm{\ }}\! +\! \frac{{{\mathrm{R}}_{\mathrm{S}}^{\mathrm{^{\prime}}}}}{{{\mathrm{T}}\! -\! \Theta }},\end{eqnarray*}

with three fitting parameters. ${{\mathrm{R}}}_0$ is the OHE coefficient due to deflection of the conduction electrons by the Lorentz force. We obtain a good fit which satisfies the Curie–Wiess law for 60 K ≤ T ≤ 300 K. The best fit (solid line in Fig. 3a) was obtained at ${{\mathrm{R}}}_0$ = −2.61 × 10^−3^cm^3^C^−1^, Θ = −66.45 K and ${\mathrm{R}}_{\mathrm{S}}^{\mathrm{^{\prime}}}$ = 0.20 cm^3^KC^−1^. ${{\mathrm{R}}}_0$ was found to be negative, which means that the OHE is dominated by electrons. This is consistent with the band structure calculations revealing that the parent NdNiO_2_ contains small electron pockets at the Fermi energy [41], which is valid even for the underdoped samples of the same doping level based on the published Hall data [3,15]. At high temperatures (>50 K), the Kondo scattering is suppressed and the AHE contribution decreases with increasing temperature, resulting in an increase in |*R_H_*| accordingly (Fig. 2a). The fitting value R_0_ corresponds to 0.12 electron per formula unit. As shown in Fig. 3b, the Hall resistivity ρ_xy_ versus magnetic field up to 35 T at temperatures from 10 K to 70 K shows no obvious deviation from linear dependence on the magnetic field (also see Supplementary Fig. S7 for the extended temperature region data). Such Curie–Weiss type temperature dependence of the positive AHE coefficient is repeatedly observed in our underdoped Nd_1−x_Sr_x_NiO_2_ infinite-layer thin films (as shown in Fig. 3c) and is also common in recently reported nickelate superconductors [3,14,15,42]. It supports the existence of free local moments at high temperatures where the Kondo scattering is negligible, and the resistivity is dominated by electron-phonon scattering. The negative values of the Weiss temperature Θ indicate AFM correlations in the localized-spin systems. Actually, a magnetic ground state is often obtained in theoretical studies [6,10,39,43,44]. A branch dispersion of magnetic excitations in undoped NdNiO_2_ has recently been revealed using RIXS [45], suggesting a spin wave of strongly coupled, antiferromagnetically aligned spins on a square lattice. A recent NMR study shows the presence of AFM fluctuations and quasi-static AFM order in Nd_0.85_Sr_0.15_NiO_2_ [46] thin film. However, the tendency toward a long-range AFM order is interrupted by the self-doping and Kondo screening effect, causing a paramagnetic state (see the positive χ, i.e. the positive AHE coefficient in Fig. 3a) as reported so far for all RNiO_2_ (R = rare-earth) parent materials [7,9]. Most recently, charge density wave states were repeatedly discovered in the parent and underdoped nickelates [47–49], which suggest a great level of similarity to cuprates. It was remarked that a charge order modulation would also disfavor the formation of long-range AFM order. In other words, the competition between the charge order and the AFM correlation might be another reason why long-range AFM order has not been observed. In a recent theoretical work, it has also been proposed that the charge order might be associated with electron transfer from Ni 3d orbitals to conduction bands close to the Fermi energy and thus provide a condition for the presence of Kondo scattering at low temperatures [50].

Based on the present findings, Fig. 3d depicts a phase diagram of the Nd_1−x_Sr_x_NiO_2_ showing a superconducting dome, combined with that in recent reports [3,15,51,52], and the characteristic temperature T*, where the *R_H_* shows a maximum. As *x* increases from the underdoped side, T* decreases monotonically and the T*−*x* curve separates the underdoped region into two parts: a Kondo scattering region and a metal. It is worth noting that the extension of the T*−*x* curve reaches the bottom of the superconducting dome (under magnetic field), which is well consistent with the recent report [52]. Thus, instead of a simple Mott insulator, the Kondo physics must also play a crucial role in nickelate superconductors. Our results may indeed have some implications on the superconductivity. Based on the Kondo mechanism in the underdoped region, our phase diagram (see Fig. 3d) suggests that superconductivity emerges near the boundary and that the Kondo effect is suppressed. As discussed previously [19], this may have an important influence on the pairing symmetry of the superconductivity. The interplay of magnetic fluctuations and Kondo hybridization could potentially lead to *d* + is pairing [19].

## CONCLUSION

Putting everything together, the logarithmic temperature dependence of resistivity and *R_H_*, the good agreement with the NRG, NCA and Hamann predictions, the linear dependence of $\sigma _{{\mathrm{xy}}}^{{\mathrm{AHE}}}{\mathrm{\ }}\sim{\mathrm{\ }}{\sigma }_{{\mathrm{xx}}}$, and the linear temperature dependence of the dephasing rate, all support the presence of magnetic Kondo scattering in the underdoped infinite-layer Nd_1−x_Sr_x_NiO_2_ thin film. According to Yang and Zhang [19], the presence of local moments cannot be ascribed to the Nd 4f moments since similar transport properties have also been observed in LaNiO_2_ [9]. The first-principles band structure calculations [41] show that the Nd 5d orbitals in NdNiO_2_ are hybridized with the Ni 3d orbitals, leading to small Fermi pockets of dominantly Nd 5d electrons in the Brillouin zone. Nd 5d conduction electrons have an electron density of n < 1 per Ni site, coupled to the localized Ni^1+^ spin-1/2 of $3{{\mathrm{d}}}_{{{\mathrm{x}}}^2 - {{\mathrm{y}}}^2}$ orbital to form Kondo spin singlets, as in the Kondo lattice systems with a small concentration of conduction electrons [20]. In conclusion, our experimental results strongly support the self-doped Mott–Kondo scenario for the underdoped Nd_1−x_Sr_x_NiO_2_ infinite-layer thin films. The present findings shed new light on the underlying physics of the infinite-layer nickelates and a possibly novel mechanism of unconventional superconductivity. It would improve our understanding of the newly discovered superconductivity in nickelates.

## Supplementary Material

nwad112_Supplemental_File
